# InterMOD: integrated data and tools for the unification of model organism research

**DOI:** 10.1038/srep01802

**Published:** 2013-05-08

**Authors:** Julie Sullivan, Kalpana Karra, Sierra A. T. Moxon, Andrew Vallejos, Howie Motenko, J. D. Wong, Jelena Aleksic, Rama Balakrishnan, Gail Binkley, Todd Harris, Benjamin Hitz, Pushkala Jayaraman, Rachel Lyne, Steven Neuhauser, Christian Pich, Richard N. Smith, Quang Trinh, J. Michael Cherry, Joel Richardson, Lincoln Stein, Simon Twigger, Monte Westerfield, Elizabeth Worthey, Gos Micklem

**Affiliations:** 1 Cambridge Systems Biology Centre, University of Cambridge, Cambridge CB2 1QR, United Kingdom; 2 Department of Genetics, University of Cambridge, Cambridge CB2 3EH, United Kingdom; 3 Department of Genetics, Stanford University, Stanford, CA, 94305, USA; 4 Ontario Institute for Cancer Research, Toronto, ON, M5G0A3, Canada; 5 The Jackson Laboratory, Bar Harbor, Maine, 04609, USA; 6 ZFIN, University of Oregon, Eugene, OR, 97405, USA; 7 Biotechnology and Bioengineering Center, Medical College of Wisconsin, Milwaukee, WI, 53226, USA; 8 Human and Molecular Genetics Center, Medical College of Wisconsin, Milwaukee, WI, 53226, USA; 9 Institute of Neuroscience, University of Oregon, Eugene, OR, 97405, USA

## Abstract

Model organisms are widely used for understanding basic biology, and have significantly contributed to the study of human disease. In recent years, genomic analysis has provided extensive evidence of widespread conservation of gene sequence and function amongst eukaryotes, allowing insights from model organisms to help decipher gene function in a wider range of species. The InterMOD consortium is developing an infrastructure based around the InterMine data warehouse system to integrate genomic and functional data from a number of key model organisms, leading the way to improved cross-species research. So far including budding yeast, nematode worm, fruit fly, zebrafish, rat and mouse, the project has set up data warehouses, synchronized data models, and created analysis tools and links between data from different species. The project unites a number of major model organism databases, improving both the consistency and accessibility of comparative research, to the benefit of the wider scientific community.

Model organism research in biological sciences is widespread and essential. From the classic cell cycle studies in yeast^1^, to the regulation of the basic body plan in *Drosophila*^2^ and a wide range of medically relevant research in rats and mice, model organisms have led to many of the major biological discoveries of the last century. The acceleration of data collection due to the technological transformation of recent years has both significantly contributed to the pool of knowledge about individual model organisms and helped confirm the belief that many of the underlying genetic principles are shared across eukaryotes^3^. This conservation of function combined with the rapidly increasing availability of new genome sequences and functional genomic data means that cross-species research promises to contribute to the molecular genetic understanding of a wide range of eukaryotic species.

Such cross-organism comparative analysis is powerful for a number of reasons. Making analogous observations in different species can strengthen evidence for a hypothesis, systematically remove gaps in knowledge, and also lead to novel findings, such as a detailed understanding of evolutionary principles and the differences between organisms^4^. Furthermore, specific model organisms have historically been used for advancing different fields of biology, and so by nature tend to provide complementary information. Uniting multiple lines of evidence promises to help the translation of model organism research to medical practice and should have a significant impact on clinical and personalized medicine, as well as health-related genomics.

Although cross-species studies are extremely powerful^5^, translating research across organisms is time consuming and requires highly specialized knowledge. Specifically, matching identifiers and coordinates across datasets, followed by collecting relevant (e.g. functional and phenotypic) data from the organisms of interest often requires collation of data from multiple different sources, specialist tools, and manual curation. One of the principal aims of the work described here is to improve the infrastructure for cross-species analysis and make analysis tools more widely accessible to researchers.

Infrastructure already exists for compiling, curating and standardizing available data for each of the model organisms in the form of freely available model organism databases (MODs). They are valuable resources to their research communities worldwide because they provide detailed levels of annotation with a particular focus on reliability and manual curation. However, although the infrastructure is highly developed within each MOD for a single organism, the fact that conventions tend to vary between the databases provides barriers to comparative analysis. Much work was therefore needed to provide data in a consistent format in combination with a unified interface and tools to facilitate consistent comparative analysis. By working together, the MODs, as existing information hubs, were ideally positioned to establish such an infrastructure with the aim of enabling easier cross-organism analysis.

Five of the major Model Organism Databases (MODs) have adopted the InterMine data integration platform to provide flexible searching and data mining interfaces to their user communities, the first time these widely used resources have converged on a common software platform (Table 1). InterMine is an open-source data warehouse system designed specifically for the integration and analysis of complex biological data^6^. It includes a web application providing a simple user interface with a set of analysis tools suitable for first time users, as well as a powerful, scriptable web-service API to allow programmatic access to data for more advanced users (for a review of the InterMine platform, see reference^6^). It was originally built for the FlyMine project^7^, and has since been used by a number of projects ranging from large-scale functional annotation of the *C. elegans* and *D. melanogaster* genomes (modENCODE^8^) drug discovery (TargetMine^9^), fruit fly transcription factors (FlyTF^10^) and mitochondrial proteomics (MitoMiner^11^).

Here we introduce and describe the progress of the InterMOD project (intermod.intermine.org), an international consortium composed of the above five model organism databases and the InterMine group. The consortium aims to make it easier to carry out cross-MOD comparative analysis and to relate MOD data to medical research. Specific goals are establishing common infrastructure, standardising terminology, implementing standardised links between the different model organisms, and creating common analysis tools.

## Results

### Data warehouse infrastructure for cross-organism research

Establishing the infrastructure for cross-organism research has required the development of web services, creation of independent embeddable web components and unification of the data model used by each of the InterMine instances. The web services allow communication between individual InterMine databases in a common language, embeddable web components promote tool and data sharing, while the unified data model ensures consistency in the data represented in each of the MOD InterMine instances and provides the basis for re-usable tools and services.

### Web services and Embeddable Web Components

InterMine is underpinned by a set of RESTful web services that give programmatic access to all core functionality. The existence of web services enables automation of comparative analysis across all existing InterMine instances as well as the development of new tools. In particular this means that tools and reports can be created that draw on data from more than one model organism InterMine database and researchers can write their own analysis pipelines utilizing any or all of the consortium databases.

The web services are platform independent, meaning that users can choose the most suitable programming platform for their needs. While any language capable of making standard HTTP requests can be used, library support is provided in five commonly used programming languages – Python, Perl, Java, JavaScript and Ruby.

Key components of the InterMine web interface (results tables, analysis tools, graphical displays) have been re-designed as organism agnostic, reusable, interactive components that can be embedded in any web site. This approach has two advantages: 1) As new tools easily can be incorporated into the existing InterMine framework, it allows developers to make their own tools and share them. In practice this means developers can now, following the documentation (http://intermine.org/embedding), develop tools based on InterMine's reusable, interactive components using the examples given, with the choice of technologies supported (JavaScript, CoffeeScript, eco, Stylus, CSS) being at the cutting edge of what is currently available. Thus, any of the MODs can create new tools that can easily be shared with all MOD InterMine instances; recently, SGD developed the YeastGenome iPhone app^12^, using web services from YeastMine^13^. 2) Any web component can be embedded within any webpage, InterMOD or not, so broadening the ways the MOD user communities can access MOD data. For example, the MODs already have established entry points for their communities, which now can embed a range of InterMine database derived results e.g. from a data mining query or a GO-term enrichment tool.

### Unifying the data model

One of the challenges that needed to be addressed was that the different MODs have different conventions for data representation. In order to facilitate querying using the same terms across databases, a shared data model is essential. For example, when querying the properties of genes, the properties need to be represented in a uniform way across the databases. As having a shared data model was essential as the foundation for cross-database operation, the development of a consistent core model was one of the first priorities of the InterMOD consortium.

The InterMine data model, or schema, is a description of the hierarchy of data types that are expected to be stored in the database. Each data type can have any number of attributes, references to other objects in the database, and collections of other objects in the database. The full model for any InterMine instance can be viewed by using the “service/model” URL extension (for example, the FlyMine model can be accessed at flymine.org/query/service/model).

**Figure 1:**
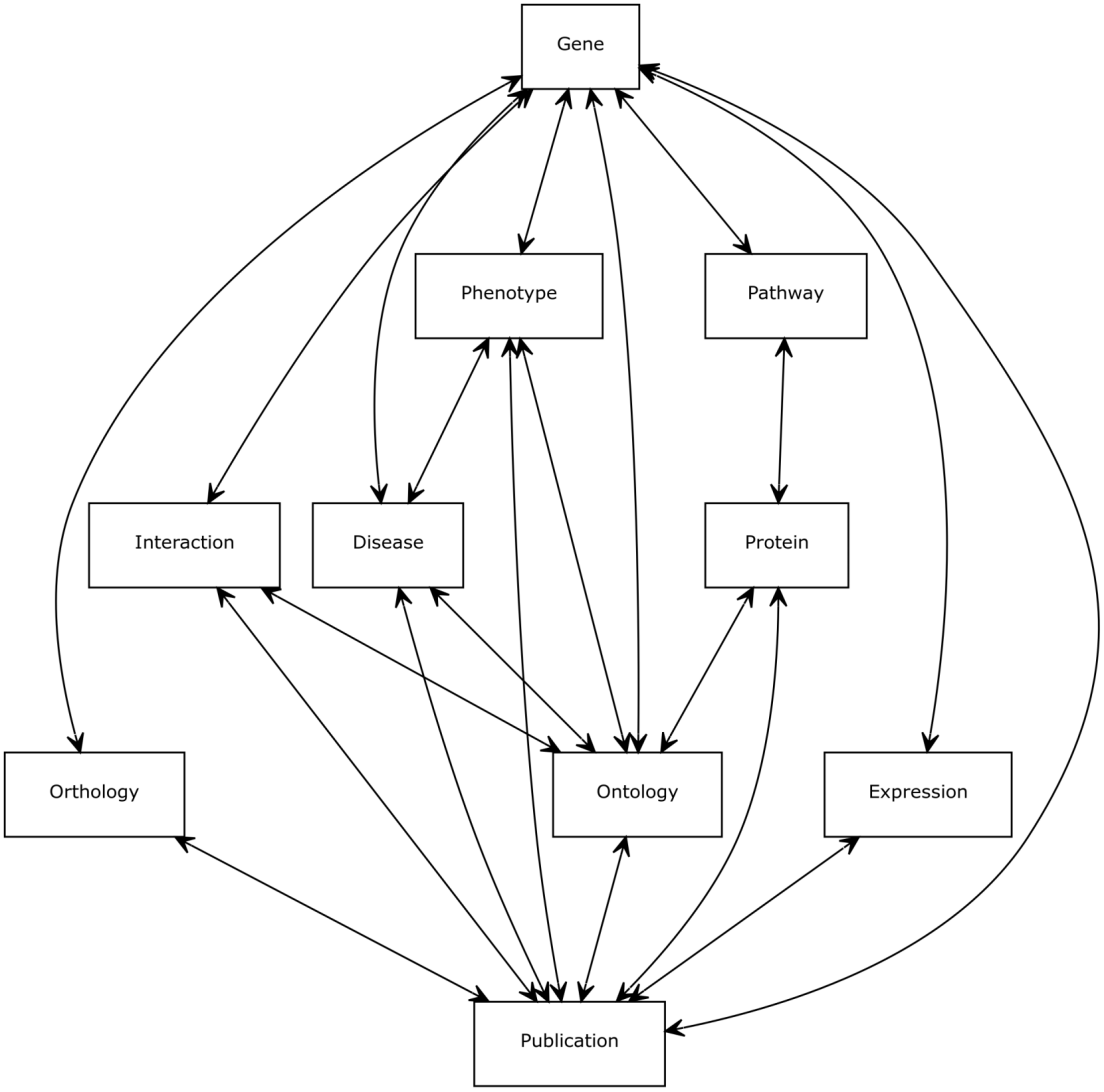
The main data types and their relationships.

The starting point for each InterMine database's data model is the InterMine Core Data Model. This subset of the full data model cannot be overridden, meaning that if a data type is included in it, this data type representation will be the same across all InterMine instances. The core model is based on the Sequence Ontology^14^ and contains biological features such as *genes* and *proteins*, related types such as *publications* as well as data types that describe data including *data sources* and *evidence codes* (Figure 1). While the core model is constant across InterMine instances, a feature of InterMine is that it is easy to extend the core to allow for organism-specific or other data types to be included. When an InterMine database is built, any additions to the model are merged into the core model and the users interface rebuilds itself to account for the changes.

To ensure the consortium data models remain as close as possible to each other, and to remove any duplication or conflicts, we provide a model comparison tool (see intermine.org/reports/models). The InterMOD Interoperability Working Group was formed with representatives of each MOD to review such comparisons and decide on which data types and fields should be part of the core model and therefore consistent between the different InterMOD InterMine databases. In case of a conflict or duplication, a standard is agreed and each InterMine instance updates their model. As a simple example, some InterMine instances had a Gene “description” field and others had a Gene “summary” field, containing essentially the same information.

We expect such data model refinements to remain an ongoing process as further tools are constructed, with the ultimate aim being to capture the major data fields relevant to cross-organism interoperation. Current efforts are focused on homology, phenotype and strain/allele data. The core model can be viewed online at github.com/intermine/intermine/blob/dev/bio/core/core.xml.

Such synchronization of the core model underpins the ability to construct cross-database queries, as the same query (e.g. requesting homologs of specified mouse genes) can be sent to any InterMine instance without modification if the data models are all the same. This also helps the MOD sites to collate related information from multiple InterMine instances and, for example, present them to their users within a single web page. In addition such uniformity helps end users: having learned how to use one consortium InterMine database, the others provide the same core data and behave similarly for both web interface and web services.

### Cross-organism analysis tools

It has required substantial time and effort to establish the core model and the working practices necessary to coordinate further extensions to it. Together with the establishment of the underlying framework of web services and embeddable web components, this unified data model has paved the way for the development of cross-species analysis tools and reports. Links between model organisms can be made through a number of different data types and in particular through orthology and ontologies as described below in more detail.

### Orthology

Orthology is a widely used basis for cross-organism analysis utilizing the shared ancestry of genes, and was thus the first linking point established between the different InterMOD data warehouses. Using orthology relies on mappings that can be difficult to define unambiguously and are the outputs of multiple significant research efforts including Panther^15^ and TreeFam^16^, as well as curation by a number of the MODs. These mappings use different underlying datasets and different methods for orthology determination. Such heterogeneity can make it difficult for researchers to know which set to use for a particular project, and the best set can vary depending on whether, for example, gene function or inferring species phylogenies is of interest. This means that there is at present no single best set of ortholog mappings for all organisms. Currently, the InterMOD project handles this by allowing each InterMine instance to use ortholog mappings selected by its host MOD. In the future we envisage supplementing these default mappings with others that can be selected by the end users. Other groups are working to create standardized ortholog mappings^17^, and the consortium will make use of them when they become available.

**Figure 2:**
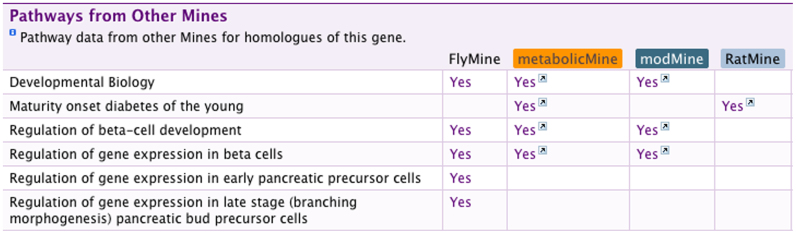
Cross species analysis of pathways. An image from part of the FlyMine report page for the *Drosophila* melanogaster forkhead gene. The rows list KEGG or Reactome pathway names. A "Yes" in the FlyMine column links to the corresponding FlyMine pathway report page. A "Yes" in the other columns indicates that an orthologue in another InterMine database shares the same pathway, with a link to the pathway report page in that database. When the web page loaded, the *Drosophila* forkhead gene was mapped to the genes *Foxa1*, *Foxa2* and *Foxa3* in rat, mouse and human, *HCM1* in yeast and *lin-31* in *C. elegans* using mappings from TreeFam. The datasets from rat (RatMine) and human (metabolicMine, unpublished) associate the orthologs with mature-onset diabetes, and highlight their role in the regulation of beta-cell development. Further pathways via FlyMine indicate that human Reactome supports roles in pancreatic cell development.

At the simplest level orthology mappings enable linking between the different InterMine instances. For example, a researcher with a list of mouse genes in MouseMine can jump to the equivalent (orthologous) rat gene list in RatMine. Such mappings also enable more detailed comparisons, such as looking for orthologous functional information. For example, a tool was developed to survey pathway annotations across organisms (Figure 2). For a given gene, this pathway tool determines the homologs and sends web service queries to the other InterMOD InterMine instances. The replies are used to create a summary of those pathways in which the genes in question are involved. This table provides an overview of gene function based on insights from different organisms. In this way it is possible for users to rapidly compare and contrast cross-species data with little effort. Furthermore, this kind of web service based cross-species process can be used to populate InterMOD summary tables or analysis tools that can be embedded in any web page, whether within the InterMOD consortium or not.

### Ontologies

Biological ontologies have been developed to provide a powerful language for describing among others, biological processes, functions, locations and sequence features. They lay the groundwork for cross-species database interoperability by allowing annotation and analysis using a set of common terms^14^,^18^. So far data using 15 different ontologies have been integrated into the different consortium InterMine databases, including, for example, Gene Ontology annotations^18^ and Anatomy Ontology mappings (Table 2). Disease ontologies^19^ are also included, and are helpful for relating model organism genes, mutations and phenotypes to human diseases. A Disease Ontology tool is already available which, for a specified gene, uses orthology to retrieve disease information from different species. As implemented in FlyMine with rat genes, this tool displays diseases and related links on gene report pages, helping *Drosophila* researchers connect basic research on a particular gene to its potential medical relevance. It is envisaged that future tools may link directly through ontology terms. However, currently, many ontologies are specific for a particular species and cannot directly be used for interoperation between species. Specialists in ontologies are developing bridging ontologies that map anatomical features in one organism to the corresponding ones in another and the same is being done for phenotypes^20^,^21^,^22^. While these efforts represent considerable challenges they will be very valuable to the InterMOD consortium in due course; one of the aims of the consortium was to anticipate the development of such mappings and it now provides a framework in which they can be exploited.

## Discussion

The InterMOD consortium has developed a framework and working practices to allow collaboration between five of the major model organism databases. Public InterMine instances are available for budding yeast, mouse, rat, and zebrafish, with the nematode worm one currently close to public beta release. A core data model essential to enable interoperation between the different databases has been established, together with a working group to review and expand the core model as needed for future developments. Orthology has been used to create a number of tools, including an ortholog exporter, a tool for obtaining disease data, and a pathway comparison tool. Work is continuing on consolidating models for gene expression, protein domains and ontologies, which will facilitate development of further tools. Improvements were also made to the InterMine infrastructure, such as expansion of web services and adding the capability for embedding analysis tools in external websites.

Having established the core model, databases, infrastructure, demonstration tools and working practices there is still much work for the consortium to do. Refinements to existing functionality include providing users with more choice in the way orthology is used for interoperation; this may include both a choice of methods, but also choice within a particular mapping. For instance, in some cases users are interested in the single best choice of ortholog and in other situations all likely orthologs are of interest^15^. Having established orthology-based interoperation the consortium is well placed to take advantage of further developments such as the efforts of the Quest for Orthologs Consortium^17^.

**Figure 3:**
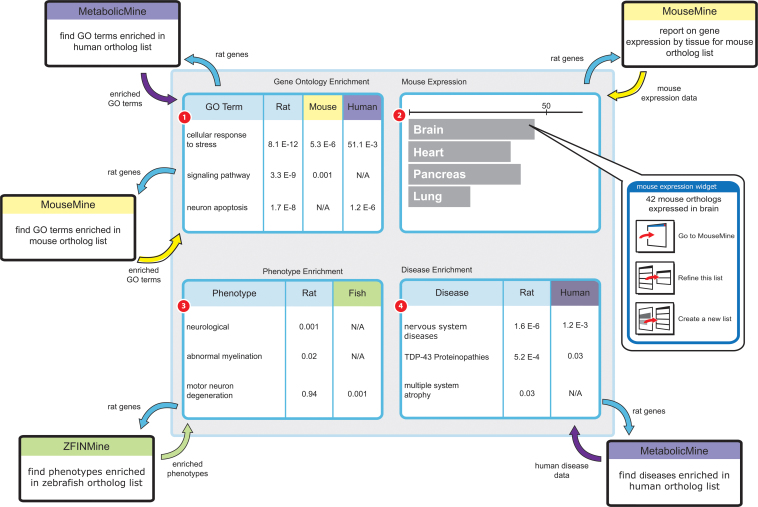
The utility of cross-species analysis. Illustration of the principle of a cross-species analysis report page using a set of rat genes linked with motor neuron degeneration, Gsn, Ppid and Brd2, and further expression, disease and phenotype data extracted from other MOD InterMine databases based on their respective orthologs. Data are drawn from mouse, human and zebrafish, to enhance the information known about the rat gene list: (1) The gene ontology enrichment can be compared and contrasted across species; (2) A tissue specific gene expression dataset is not available in the rat database, so data from mouse are used instead, to complement the analysis; (3) Data from zebrafish provide further evidence for a motor neuron degeneration phenotype, and for cross-species conservation of gene function related to this phenotype; (4) A disease ontology comparison tool provides a direct link to human disease and data.

Other kinds of mappings, such as synteny, are also of interest. Providing regions of shared synteny will allow researchers to compare features such as regulatory regions and transcription factor binding profiles across species. Similarly, refinement of the core model will facilitate development of tools for cross-species gene expression and protein interaction network analysis. Potentially, a large range of cross-species information can be displayed on interactive report pages (such as the one illustrated in Figure 3), with InterMOD providing the foundations and building some of the associated analysis tools.

Further work to support ontologies is likely to be another future area of work for the consortium. The MODs collectively have been major developers and consumers of the leading ontologies, and the InterMOD consortium is providing new ways of exploiting these ontologies. Developments underway^20^,^21^,^22^ to provide cross-species ontologies that provide mappings between anatomical terms and phenotypes in one organism to the corresponding ones in another should greatly increase the power and flexibility of cross-species analysis that can be carried out by non-bioinformaticians. The framework that has been put in place means that the InterMOD consortium is in an excellent position to draw on these and any other ontologies that emerge.

A greater range of organisms can be added to the project; although many of the major model organisms are already represented, the consortium is open to further collaborators. Additional organisms may provide further synergy. For instance, the InterMine team has secured funding to develop HumanMine, a large data warehouse of human genetic, genomic and proteomic data that will extend the existing metabolicMine database (metabolicmine.org). HumanMine should provide a common interoperation stage for the other InterMOD databases, so enhancing translational research by enabling a uniform way for model organism researchers to relate their findings to human data, as well as making it easier for medical researchers to access relevant model organism data.

Apart from interoperation, there is a clear advantage in unifying model organism data using a common platform in that it reduces the time and effort required to learn many different user interfaces. For example, a researcher working in yeast biology can repeat analyses on zebrafish or fly data without further training, benefit from similar tools and retrieve data in a consistent format.

In conclusion, the InterMOD consortium has built a number of organism-specific InterMine data warehouses using a shared data framework, and establishing cross-species interoperation between them. The consortium has drawn on previous work such as the Gene and Sequence Ontologies, and anticipates the maturation of further ontologies such as phenotype and anatomical ontologies. The collaboration between the InterMine team and model organism databases within this consortium promises a platform for efficient cross-species comparative analysis. It also provides the potential for further synergies in quality checking and building common tools for the benefit of the broader biomedical research community. Importantly, the links that will be established to a core set of human data will provide greater opportunities for medical research to benefit from model organism findings, as well as providing the framework of cross-species validation valuable for translating model organism research into medical applications.

## Methods

### Software details

All data warehouses for the different model organisms were set up using the open-source InterMine platform (version 1.1). InterMine is available under an LGPL licence (http://www.gnu.org/licenses/lgpl.html), and the code and documentation can be obtained from http://www.intermine.org, along with links to the different existing InterMine databases. The technical details of the InterMine framework, its associated software dependencies and comparison to other systems have been previously described^6^. All of the MODs developed and maintain their own InterMine databases. See Table 1 for a list of the MODs that are part of the InterMOD consortium, together with URLS for the MOD and MOD InterMine databases.

### Data model consistency checks

The essential core data model is being expanded after initial efforts focused on gene and homolog datatypes. These datatypes highlight the types of consistency checks that have to be carried out. It is important that field names are both named consistently and contain equivalent information. For example, the fields in which key gene identifiers are held must be consistent between the databases. Several common fields were agreed upon for the “gene” data type: primary identifier, secondary identifier, symbol and name. All of these except for the primary identifier can be empty and non-unique. Once the basic types had been agreed upon, a standard protocol was set up for selecting the data identifiers, with other existing identifiers saved as cross-references. Specifically, the MOD-assigned IDs (e.g. ZDB-GENE, MGI:, RGD:) are the primary identifiers, while other identifiers (e.g. Ensembl, RefSeq) are saved as cross references. NCBI identifiers are used in the case of human genes. For historical reasons, some MODs have more than one identifier for each gene. In these cases the second MOD identifier is stored in the “secondary identifier” column. In earlier databases, some InterMine instances had specific fields to hold cross-references, e.g. “RefSeq identifier” or “GenBank identifier”. However, given the sheer number of external databases in existence, and the fact that many hold multiple identifiers for the same gene, it was considered too unwieldy to create a specific field for each external database. Therefore, to ensure consistency, all external identifiers are now stored as a set of “cross-references”.

Another example where significant model-reconciliation needed to be addressed was with “sequence features”- i.e. how the InterMine databases provide information about what a particular sequence feature is. In addition to different field names for feature types, specifically, “feature type”, “gene type” and “type”, there were also differences in the contents of the fields, with some using their own terms while others used terms defined by the Sequence Ontology. During the data-loading phase every sequence feature in InterMine automatically is assigned a term from the Sequence Ontology based on the feature type and this is stored as a Sequence Ontology term referenced from Gene. For example, every gene in the database has a reference to the Sequence Ontology term “gene” (SO:0000704). To synchronize both the data model and the terminology, it was agreed to remove all type-related fields from the data model of each InterMine instance and use this reference to the SO term instead.

### Data linking points

Each InterMine instance uses a default mapping for orthologous genes, which are then used by the analysis tools, for example to compare pathways. If no local ortholog mappings are available, the genes are sent to the other linked InterMine databases, and the orthologs are mapped using the local mappings available in those InterMine instances. InterMine web services are used exchange searches and results between the different InterMine databases.

The choice of mappings varies between different InterMine instances, with the choice of default mapping determined by the coverage offered by the different mappings for the model organism in question. The homolog data parsers available from the InterMine library include TreeFam^16^, Ensembl^23^, OrthoDB^24^, Homologene^25^, InParanoid^26^ and Panther^15^. The MODs have also written custom data parsers using the APIs to load their own curated homolog data. Currently, FlyMine and YeastMine use Treefam mappings^16^, MouseMine uses Homologene^25^. ZFINMine loads the ZFIN curated data, and RatMine loads both RGD and MGI curated data.

### Pathway comparison

The pathway data is imported from sources including KEGG^27^, Reactome^28^ and manually curated pathway data from individual MODs. The pathway analysis tool (shown in Figure 2) first queries the local InterMine instance for homologs. If homologs are found, these are sent to the linked InterMine instances, and if none are found the original identifiers are sent instead, to be converted at the remote InterMine. A query is then run in the linked InterMine databases to determine which pathways are associated with the list of genes in question. The result is then displayed on the gene report page of the InterMine database that initiated the search.

## Author Contributions

Model organism-specific InterMine instance development and maintenance: H.M., S.N. and J.R. for MouseMine; A.V., P.J., S.T. and E.W. for RatMine; J.D.W., T.H., Q.T. and L.S. for WormMine; K.K., R.B., G.B., B.H. and J.M.C. for YeastMine; S.A.T.M., C.P. and M.W. for ZFINMine; J.S., R.L., J.A., R.N.S. and G.M. for FlyMine; J.S. coordinated the project and provided developer support. G.M. conceived and managed the project. J.A., J.S., R. L., J.M.C., M.W. and G.M. drafted the manuscript, with J.A. as lead drafter. All authors read and approved the final manuscript.

## Figures and Tables

**Table 1:** Model organism databases involved with the InterMOD consortium

Model organism databases	Organism	MOD URL	MOD InterMine Database URL
Mouse Genome Informatics^29^	Mouse	informatics.jax.org	www.mousemine.org
Rat Genome Database^30^	Rat	rgd.mcw.edu	ratmine.mcw.edu
Saccharomyces Genome Database^13^	Budding yeast	yeastgenome.org	yeastmine.yeastgenome.org
WormBase^31^	Nematode worm	wormbase.org	intermine.wormbase.org [summer 2013]
ZFIN^32^	Zebrafish	zfin.org	zmine.zfin.org/zebrafishmine

**Table 2:** A list of ontologies loaded into InterMine participating in the InterMOD project

Ontology	Mine
Cell Type^33^	RatMine
Disease^19^	RatMine
Fly Anatomy^34^	FlyMine
Fly Development^34^	FlyMine
Gene^18^	FlyMine, MouseMine, RatMine, YeastMine, ZFINMine
Mammalian Phenotype^35^	MouseMine, RatMine
MEDIC^36^	MouseMine
Mouse Adult Gross Anatomy^37^	RatMine
PATO^26^	ZFINMine
Pathway^38^	RatMine
PSI Molecular Interactions^39^	FlyMine
Rat Strain^40^	RatMine
Sequence^14^	FlyMine, MouseMine, RatMine, YeastMine, ZFINMine
Uber Anatomy Ontology^20^	FlyMine
ZFIN anatomy^41^	ZFINMine
